# Impact of Morbid Obesity and Obesity Phenotype on Outcomes After Transcatheter Aortic Valve Replacement

**DOI:** 10.1161/JAHA.120.019051

**Published:** 2021-05-31

**Authors:** Angela McInerney, Gabriela Tirado‐Conte, Josep Rodes‐Cabau, Francisco Campelo‐Parada, Jose D. Tafur Soto, Marco Barbanti, Erika Muñoz‐Garcia, Mobeena Arif, Diego Lopez, Stefan Toggweiler, Gabriela Veiga, Anna Pylko, Teresa Sevilla, Miriam Compagnone, Ander Regueiro, Viçent Serra, Manuel Carnero, Juan F. Oteo, Fernando Rivero, Henrique Barbosa Ribeiro, Leonardo Guimaraes, Anthony Matta, Natalia Giraldo Echavarria, Roberto Valvo, Federico Moccetti, Antonio J. Muñoz‐Garcia, Javier Lopez‐Pais, Bruno Garcia del Blanco, Diego Carter Campanha Borges, Eric Dumont, Nieves Gonzalo, Enrico Criscione, Maciej Dabrowski, Fernando Alfonso, Jose M de la Torre Hernández, Asim N. Cheema, Ignacio J. Amat‐Santos, Francesco Saia, Javier Escaned, Luis Nombela‐Franco

**Affiliations:** ^1^ Cardiovascular Institute Hospital Clínico San Carlos Instituto de Investigación Sanitaria San Carlos Madrid Spain; ^2^ Quebec Heart and Lung Institute Laval University Quebec City Quebec Canada; ^3^ Cardiology Department Rangueil University Hospital Toulouse France; ^4^ The Ochsner Clinical School Ochsner Medical Center New Orleans LA; ^5^ Ferrarotto Hospital University of Catania Catania Italy; ^6^ Centro de Investigación Biomédica en Red Enfermedades Cardiovasculares Cardiology Department Hospital Universitario Virgen de la Victoria Málaga Spain; ^7^ Division of Cardiology St. Michael’s Hospital Toronto University Toronto Ontario Canada; ^8^ Hospital Clínico Universitario de Santiago CIBERCV Santiago Spain; ^9^ Heart Center Lucerne Luzerner Kantonsspital Lucerne Switzerland; ^10^ Hospital Universitario Marques de Valdecilla IDIVAL Santander Spain; ^11^ Department of Interventional Cardiology and Angiology National Institute of Cardiology Warsaw Poland; ^12^ CIBERCV Instituto de Ciencias del Corazón Hospital Clínico Universitario de Valladolid Valladolid Spain; ^13^ Cardiology Unit Cardio‐Thoracic‐Vascular Department University Hospital of Bologna Policlinico S, Orsola–Malpighi Bologna Italy; ^14^ Cardiology Department Cardiovascular Institute, Hospital Clínic Universidad de Barcelona Institut d'Investigacions Biomèdiques August Pi i Sunyer Barcelona Spain; ^15^ Hospital General Universitari Vall d’Hebron Barcelona Spain; ^16^ Department of Cardiology Hospital Universitario Puerta de Hierro Majadahonda Spain; ^17^ Cardiology Department Hospital Universitario de la Princesa Instituto de Investigación Sanitaria Princesa (IIS‐IP) Universidad Autónoma de Madrid, CIBER‐CV Madrid Spain; ^18^ Heart Institute (Instituto de Coraçāo) Sao Paulo Brazil

**Keywords:** epicardial adipose tissue, morbid obesity, subcutaneous adipose tissue, transcatheter aortic valve replacement, visceral adipose tissue, Catheter-Based Coronary and Valvular Interventions, Obesity, Valvular Heart Disease

## Abstract

**Background:**

There is a paucity of outcome data on patients who are morbidly obese (MO) undergoing transcatheter aortic valve replacement. We aimed to determine their periprocedural and midterm outcomes and investigate the impact of obesity phenotype.

**Methods and Results:**

Consecutive patients who are MO (body mass index, ≥40 kg/m^2^, or ≥35 kg/m^2^ with obesity‐related comorbidities; n=910) with severe aortic stenosis who underwent transcatheter aortic valve replacement in 18 tertiary hospitals were compared with a nonobese cohort (body mass index, 18.5–29.9 kg/m^2^, n=2264). Propensity‐score matching resulted in 770 pairs. Pre–transcatheter aortic valve replacement computed tomography scans were centrally analyzed to assess adipose tissue distribution; epicardial, abdominal visceral and subcutaneous fat. Major vascular complications were more common (6.6% versus 4.3%; *P*=0.043) and device success was less frequent (84.4% versus 88.1%; *P*=0.038) in the MO group. Freedom from all‐cause and cardiovascular mortality were similar at 2 years (79.4 versus 80.6%, *P*=0.731; and 88.7 versus 87.4%, *P*=0.699; MO and nonobese, respectively). Multivariable analysis identified baseline glomerular filtration rate and nontransfemoral access as independent predictors of 2‐year mortality in the MO group. An adverse MO phenotype with an abdominal visceral adipose tissue:subcutaneous adipose tissue ratio ≥1 (VAT:SAT) was associated with increased 2‐year all‐cause (hazard ratio [HR], 3.06; 95% CI, 1.20–7.77; *P*=0.019) and cardiovascular (hazard ratio, 4.11; 95% CI, 1.06–15.90; *P*=0.041) mortality, and readmissions (HR, 1.81; 95% CI, 1.07–3.07; *P*=0.027). After multivariable analysis, a (VAT:SAT) ratio ≥1 remained a strong predictor of 2‐year mortality (hazard ratio, 2.78; *P*=0.035).

**Conclusions:**

Transcatheter aortic valve replacement in patients who are MO has similar short‐ and midterm outcomes to nonobese patients, despite higher major vascular complications and lower device success. An abdominal VAT:SAT ratio ≥1 identifies an obesity phenotype at higher risk of adverse clinical outcomes.

**Nonstandard Abbreviations and Acronyms**
EAT: epicardial adipose tissue
IMAT: intramuscular adipose tissue
iVAT: indexed visceral adipose tissue
MO: morbidly obese
MVC: major vascular complication
PPM: patient‐prosthesis mismatch
SAT: subcutaneous adipose tissue
TAVR: transcatheter aortic valve replacement
VAT: visceral adipose tissue

**Clinical Perspective**

**What Is New?**

- The number of patients who are morbidly obese undergoing transcatheter aortic valve replacement is increasing, but a paucity of data regarding their outcomes remains.
- Patients who are morbidly obese undergoing transcatheter aortic valve replacement have similar in‐hospital, 30‐day, and 2‐year mortality to their nonobese counterparts.
- Major vascular complications are, however, increased in the morbidly obese population and device success is lower, mainly driven by increased mean valve gradients and patient‐prosthesis mismatch; adipose tissue distribution analysis using the pre–transcatheter aortic valve replacement computed tomography scans can identify a population of patients who are morbidly obese with an adverse obesity phenotype (visceral adipose tissue:subcutaneous adipose tissue ratio ≥1) and increased risk of mortality at 2 years.

**What Are the Clinical Implications?**

- Patients who are morbidly obese can safely be offered transcatheter aortic valve replacement as a treatment for severe aortic stenosis.
- Vigilance is required when performing vascular access in patients who are morbidly obese who have an increased risk of major vascular complications.
- Adipose tissue distribution can identify an adverse obesity phenotype and provide important prognostic information beyond that of body mass index alone, which may avoid futile procedures in patients who are morbidly obese.

Worldwide, the obesity epidemic continues to grow. In 2016, the World Health Organization estimated that 650 million people worldwide were obese, with projections in the United States suggesting that 1 in 4 adults will have severe obesity by 2030.^1^, ^2^ Coupled with this, our aging population has resulted in a concomitant increase in the number of patients who are obese presenting with severe aortic stenosis and undergoing both surgical and transcatheter aortic valve replacement (TAVR). A 25‐fold increase in the rate of patients who are obese undergoing TAVR in the United States between 2011 and 2014 has been reported,^3^ reflecting the rapidly expanding indications for TAVR encompassing patients from low to high surgical risk profiles.^4^ However, patients who are obese, and in particular patients who are morbidly obese (MO), are underrepresented in clinical trials, with a paucity of data regarding the long‐term outcomes after TAVR in this specific population.

Patients who are MO present a unique set of challenges, with many presenting with metabolic syndrome–related comorbidities and respiratory and mobility problems, which may make their periprocedural recovery more complex. However, within the domain of cardiovascular disease, the presence of an “obesity paradox” continues to be debated, although recently, this paradigm has been challenged in patients who are MO.^5^, ^6^ In the TAVR field, conflicting results exist concerning the periprocedural and long‐term outcomes in patients who are obese.^7^, ^8^, ^9^, ^10^, ^11^, ^12^, ^13^ Interpretation of these data is further hampered by small patient numbers, particularly in the MO group,^14^ involving mainly single centers, and heterogeneity of body mass index (BMI) cutoff points used to define patient categories. Furthermore, the use of BMI is a relatively crude marker of obesity being unable to differentiate between fat mass and muscle mass. Given these limitations, there is an increasing interest in the use of an obesity or metabolic phenotype to more clearly differentiate metabolically healthy from unhealthy patients who are obese rather than simply using BMI alone.^15^ Computed tomography (CT) scans can be used to assess a patient’s obesity phenotype and assess the distribution of adipose tissue components. The objectives of our study, therefore, were (1) to compare periprocedural complications and midterm outcomes in a large cohort of patients who are MO, both unmatched and matched to a nonobese cohort; (2) to determine prognostic factors among patients who are MO after TAVR; and (3) to evaluate the predictive value of adipose tissue distribution on clinical outcomes in patients who are MO.

## Methods

Requests to access the data set, analytic methods, and study materials may be sent to the corresponding author. This was a multicenter study collecting individual data on consecutive patients who are MO with symptomatic severe aortic stenosis who underwent TAVR in 18 tertiary care centers from Europe and the United States. In addition, 6 centers provided full data on their entire nonobese TAVR cohort as a comparator group. The decision to perform TAVR was made at each center as per their local protocol, and TAVR was performed as previously described.^16^ The study was performed in accordance with the institutional review board of participating centers, and all patients provided informed consent for the procedures. All procedural‐related aspects were at the operator’s discretion.

BMI was calculated as weight in kilograms/height in meters squared. The following definitions were applied to define the study groups such that the nonobese group included patients with a BMI of 18.5 to 29.9 kg/m^2^, and the MO group included patients with a BMI ≥40 kg/m^2^, or ≥35 kg/m^2^ with obesity‐related comorbidities.^17^, ^18^ Patients with a BMI <18.5 kg/m^2^ or 30 to 34.9 kg/m^2^ were excluded. Baseline, periprocedural, and follow‐up clinical data were prospectively collected in a dedicated database in each participating center and the coordinating center performed the statistical analysis. Periprocedural events were defined using the Valve Academic Research Consortium‐2 criteria, including device success and patient‐prosthesis mismatch (PPM) with differing cutoff points for obese and nonobese groups as specified.^19^ To determine the presence of PPM, previously defined predicted effective orifice area for each valve type and size were used.^20^ Clinical follow‐up was at 30 days, 12 months, and yearly thereafter. Midterm outcomes were assessed at 24 months.

### CT Analysis of Adipose Tissue Distribution

**Figure 1:**
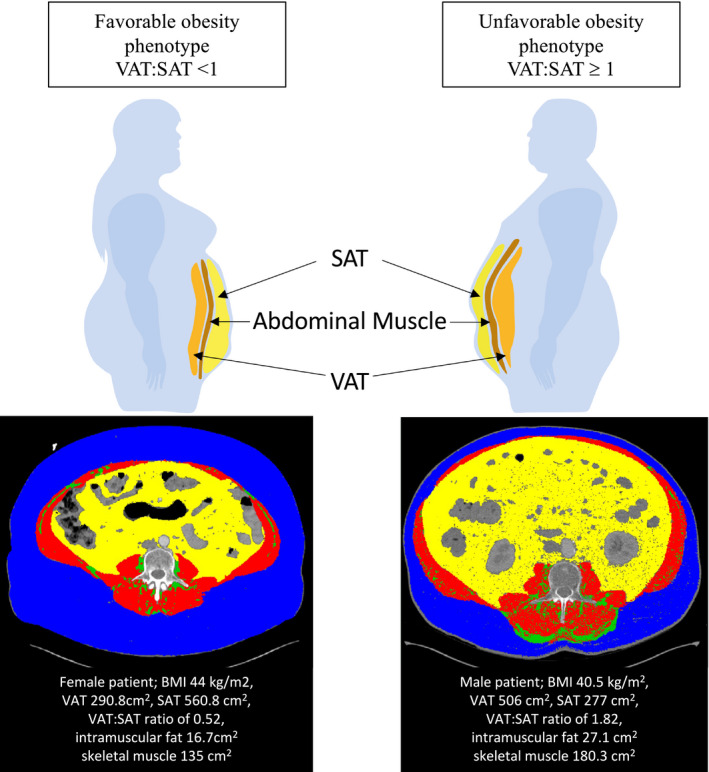
Favorable and unfavorable obesity phenotype. Abdominal adipose tissue distribution from a single computed tomography slice is shown with subcutaneous (blue) and visceral (yellow) adipose tissue, separated by the abdominal muscular layer (red). Intramuscular adipose tissue is seen in green. Favorable metabolic phenotype is seen in a female patient with a BMI of 44 kg/m^2^ and a visceral to subcutaneous adipose tissue ratio (VAT:SAT ratio) of 0.52. Unfavorable metabolic phenotype is seen in a male patient with a BMI of 40.5 kg/m^2^ and a VAT:SAT ratio of 1.82. BMI indicates body mass index; SAT, subcutaneous adipose tissue; and VAT, visceral adipose tissue.

An adipose tissue distribution substudy was performed on baseline pre‐TAVR CT scans. CT images from patients who are MO were centrally analyzed in a core laboratory at the coordinating center using a specific software package (sliceOmatic version 5.0; TomoVision, Quebec, Canada). Two separate analyses were performed. First, an analysis of abdominal fat components on 2 consecutive cross‐sectional slices taken at the third lumbar spine as previously described.^21^, ^22^ The software package was used to segment each slice into its various tissue components based on Hounsfield units in a semiautomatic fashion. Adipose tissue was measured using a Hounsfield unit threshold between −190 and −30, while muscle was set as Hounsfield units between −29 and +150. Visceral (VAT) and subcutaneous (SAT) adipose tissue were separated by the borders of the abdominal muscles (Figure 1). Intramuscular adipose tissue (IMAT) was considered to be any fat contained within the muscle capsule. Areas (centimeters squared) of VAT, SAT, and IMAT, and skeletal muscle area (SMA) were measured and recorded for each slice. The measurements from the 2 third lumbar spine slices were averaged and all results were indexed to body surface area (iVAT, indexed SAT, indexed IMAT, indexed SMA; cm^2^/m^2^). An analysis of the effect of sarcopenic obesity was also performed on the basis of previously defined sex‐specific SMAs indexed to height squared (height^2^) (rather than body surface area). Using this definition, height‐indexed SMA ≤38.5 cm^2^/m^2^ for women and ≤52.5 cm^2^/m^2^ for men were considered sarcopenic.^23^ The proportion of VAT compared with SAT was calculated by the VAT:SAT ratio and percentage VAT [(VAT/(VAT+SAT))*100] as previously described.^24^ A second analysis of epicardial adipose tissue (EAT), defined as all fat contained within the fibrous pericardium from the bifurcation of the pulmonary artery to the diaphragm,^25^ was performed on contrast cardiac CT studies. A Hounsfield unit range of −190 to −30 was used.^25^ EAT was manually redefined every 3 slices, with interpolation of intervening slices. The software calculated the EAT volume (centimeters cubed) by summing the EAT area in each slice and taking into account slice thickness and intersection gap (Figure S1 and Video S1). Results were also indexed to body surface area (centimeters cubed/meters squared).

### Statistical Analysis

Categorical variables were expressed as number and percentage while continuous variables were expressed as mean and SD or median and interquartile range (25th–75th percentile) according to their distribution. Normality was assessed using the Kolmogorov‐Smirnov text. Qualitative variables were analyzed using the χ^2^ or the Fisher exact test and differences in continuous variables using a 2‐sided *t* test or Wilcoxon rank test for the unmatched comparison. A propensity score–matched analysis was also performed between the 2 groups. A propensity score was estimated using a logistic regression model. Morbid obesity was the dependent variable, and independent variables were those baseline characteristics found to have statistically significant differences between obesity groups and other variables considered to be clinically relevant. The final variables included in the propensity matching were age, sex, diabetes mellitus, coronary artery disease, chronic obstructive pulmonary disease, peripheral vascular disease, and femoral access. A propensity score–matched cohort was then created with a 1:1 ratio of nonobese patients and patients who were MO using a “nearest neighbor” match without replacement. A caliper of <0.1× the SD of the logistic score was applied. Standardized differences were calculated for all covariates before and after matching and represented graphically in density plots to assess balance (Figure S2). Comparison of continuous and categorical variables between the matched groups were as previously stated for unmatched groups. Freedom from mortality and readmission curves were calculated using the Kaplan‐Meier method and compared using the log‐rank test in both the unmatched and matched cohorts. Cox regression analysis was used in the whole unmatched cohort to determine if morbid obesity (as a dichotomous variable) or BMI (as a continuous variable) were associated with 2‐year all‐cause mortality. Cox regression analysis was then repeated exclusively in the patients who were MO to further evaluate independent predictive factors for all cause 2‐year mortality in the MO group. Proportionality hazard assumption for Cox models was tested using Schoenfeld residuals. Testing for influential observations was with the DFBETA index. Adipose tissue measurements were additionally analyzed to investigate their impact on all‐cause and cardiovascular mortality and readmission rates. All data were analyzed with Stata version 15.1 (StataCorp, College Station, TX).

## Results

### Patient Population

Thirty‐four (3.6%) patients with a BMI of 35 to 39.9 kg/m^2^ not meeting the MO definition^17^, ^18^ were not included in the analysis. Finally, a total of 3174 patients undergoing TAVR were included; 2264 in the nonobese group and 910 in the MO group. Baseline characteristics of the unmatched population are summarized in Table 1. Patients who were MO were younger (76.8 versus 81.7 years), more likely women (66.8% versus 50.3%), with lower surgical risk scores (median Euroscore II 3.58 versus 4.10; *P*=0.001). Procedural aspects of the unmatched cohorts are summarized in Table S1. Vascular access was predominantly transfemoral (≈87%) in both groups with fewer patients who are MO having general anesthesia.

**Table 1:** Baseline Characteristics in Nonobese and Morbidly Obese Cohorts

	Unmatched Cohort			Matched Cohort		
	BMI 18.5–29.9 (n=2264)	BMI >35 (n=910)	*P* Value	BMI 18.5–29.9 (n=770)	BMI >35 (n=770)	*P* Value
Age, y	81.7 (6.4)	76.8 (7.4)	<0.001	78.1 (7.4)	78.5 (6.2)	0.206
Female sex	1138 (50.3)	608 (66.8)	<0.001	473 (61.4)	487 (63.3)	0.462
Body mass index, kg/m^2^	25.7 (2.7)	39.5 (5.2)	…	25.9 (2.7)	38.9 (4.2)	…
Diabetes mellitus	659 (29.1)	496 (54.5)	<0.001	388 (50.4)	377 (49)	0.575
Insulin use	100 (24.6)	187 (40.9)	<0.001	59 (25.3)	133 (38.1)	0.001
Hypertension	1845 (81.5)	850 (93.4)	<0.001	635 (82.5)	727 (94.4)	<0.001
Hyperlipidemia	1117 (59.9)	652 (74.7)	<0.001	443 (64.2)	533 (72.6)	0.001
Baseline creatinine, mg/dL	1.24 (0.79)	1.26 (0.86)	0.545	1.22 (0.87)	1.26 (0.84)	0.398
eGFR <30 mL/min/1.73 m^2^	177 (7.8)	79 (8.7)	0.419	58 (7.5)	64 (8.3)	0.571
Coronary artery disease	1017 (44.9)	391 (43)	0.312	349 (45.3)	328 (42.6)	0.281
Previous myocardial infarction	295 (13.1)	111 (12.3)	0.551	132 (17.2)	92 (12.0)	0.004
Previous PCI	563 (24.9)	228 (25.1)	0.904	186 (24.2)	189 (24.6)	0.859
Previous CABG	173 (7.7)	111 (12.2)	<0.001	71 (9.2)	94 (12.2)	0.060
Previous valve surgery	116 (5.1)	57 (6.3)	0.199	45 (5.9)	45 (5.9)	0.995
Valve‐in‐valve TAVR	103 (4.6)	50 (5.9)	0.160	41 (5.4)	41 (5.7)	0.800
Atrial fibrillation	743 (32.9)	317 (34.9)	0.269	257 (33.4)	268 (34.9)	0.542
Previous pacemaker	222 (9.8)	82 (9.1)	0.556	67 (8.7)	77 (10.0)	0.381
COPD	456 (20.2)	264 (29.0)	<0.001	210 (27.3)	194 (25.2)	0.354
Previous cerebrovascular accident	291 (12.9)	100 (11)	0.146	102 (13.3)	91 (11.8)	0.392
Peripheral vascular disease	362 (16.0)	111 (12.2)	0.006	108 (14.0)	101 (13.1)	0.602
NYHA functional class III and IV	1198 (53.1)	663 (73.0)	<0.001	485 (63.3)	547 (71.2)	0.001
Baseline hemoglobin, g/dL	12.0 (1.7)	12.0 (1.6)	0.615	11.8 (1.6)	12.0 (1.6)	0.050
NT‐proBNP	2154 [840–5163]	1133 [380–2800]	<0.001	1758.5 [577.5–4197]	1183 [428 –2902]	0.002
Logistic EuroSCORE	13.64 [9.12–22.34]	11.13 [7.01–18.66]	<0.001	12.91 [8.225–21]	11.59 [7.79–19.39]	0.037
EuroSCORE II	4.1 [2.54–6.86]	3.58 [2.16–5.77]	0.001	4.135 [2.5–7.07]	3.6 [2.20–5.95]	0.011
STS	4.7 [3.2–7.034]	4 [2.725–6.104]	<0.001	4.6 [2.96–6.951]	4 [2.765–6.258]	<0.001
Echocardiographic data						
LVEF	56.3 (14)	57.1 (11.2)	0.138	56.6 (14.5)	57.1 (11.1)	0.459
Mean aortic gradient, mm Hg	45 [37–56]	46 [39–56]	0.180	45 [36–55]	46 [39–55]	0.273
Aortic valve area, cm^2^	0.68 (0.23)	0.73 (0.20)	<0.001	0.67 (0.24)	0.73 (0.21)	<0.001
Moderate or severe mitral regurgitation	397 (18.9%)	135 (16%)	0.210	152 (20.0%)	112 (15.7%)	0.030
Moderate or severe aortic regurgitation	274 (12.3%)	96 (11.3%)	0.454	100 (13.2%)	83 (11.6%)	0.351
Moderate or severe PHT	638 (36.0%)	384 (48.2%)	<0.001	270 (43.1%)	316 (47.5%)	0.114

Values are expressed as mean (SD), median [IQR] or n (%). BMI indicates body mass index; CABG, coronary artery bypass grafting; COPD, chronic obstructive pulmonary disease; eGFR, estimated glomerular filtration rate; LVEF, left ventricular ejection fraction; NT‐proBNP, N‐terminal pro‐B‐type natriuretic peptide; NYHA, New York Heart Association; PCI, percutaneous coronary intervention; PHT, pulmonary hypertension; STS, Society of Thoracic Surgeons; and TAVR, transcatheter aortic valve replacement.

After propensity score application, a matched cohort with 770 patients per group was obtained. Although close matching was achieved (Figure S2A and B), some baseline cardiovascular risk factors, such as hypertension, hyperlipidemia, and insulin‐requiring diabetes mellitus, continued to differ between groups (Table 1). Most procedural aspects in the matched group were similar, although general anesthesia was still less common in patients who were MO (Table 2). Patients who were MO were also more likely to have larger‐sized valves implanted (34.1% versus 28.6% for valve sizes 29–34 mm; *P*=0.020), with less postdilation compared with the nonobese group.

**Table 2:** Procedural Aspects of Propensity Score–Matched Nonobese and Morbidly Obese Cohorts

	BMI 18.5–29.9 (n=770)	BMI >35 (n=770)	*P* Value
Procedural urgency			
Urgent/emergent procedure	63 (9.1)	50 (7.3)	0.222
Access site			
Transfemoral	684 (88.8)	670 (87.0)	0.274
Nontransfemoral	86 (11.17)	100 (12.99)	0.274
Method of transfemoral access* ^,^ ^†^			
Percutaneous with closure device	550 (86.5)	584 (90.7)	0.018
Surgical cutdown	86 (13.5)	60 (9.3)	0.018
Prosthesis type			
BEV	332 (43.1)	353 (45.8)	0.282
SEV	438 (56.9)	410 (53.3)	0.151
Edwards Sapiens & XT & S3	332 (43.1)	353 (45.8)	0.282
Medtronic Corevalve, Evolut R, Evolut Pro	363 (47.1)	347 (45.1)	0.413
Other (Portico, accurate neo, other)	75 (9.7)	70 (9.1)	
Prosthesis size			
20–23 mm	199 (26)	170 (22.2)	0.083
25–27 mm	346 (45.4)	328 (43.40)	0.442
29–34 mm	218 (28.6)	258 (34.1)	0.020
General anesthesia	354 (46)	288 (37.4)	0.001
Prior balloon valvuloplasty	454 (63)	418 (60)	0.246
Balloon post‐dilatation	151 (20.4)	93 (12.3)	<0.001

Values are expressed as n (%). BEV indicates balloon expandable valve; BMI, body mass index; and SEV, self‐expanding valve.

* One center that practices only “cutdown” technique for femoral access was excluded.

^†^ Femoral access only.

### In‐Hospital and Midterm Outcomes

**Figure 2:**
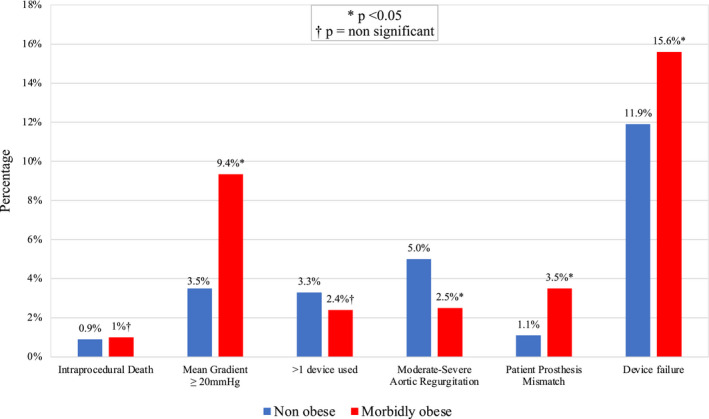
Chart depicting the causes of device failure among the matched nonobese patients vs patients who are morbidly obese.

Table S2 and Table 3 summarize periprocedural complications in the unmatched and matched populations, respectively. Major vascular complications (MVCs) occurred more commonly in the MO group (unmatched, 6.6% versus 4.6%, *P*=0.019; and matched 6.6% versus 4.3%, *P*=0.043). No differences in bleeding, hospital‐acquired pneumonia, and acute kidney injury, were noted. Device success was lower in the MO group (unmatched, 83.4% versus 89.7%, *P*=0.001; and matched 84.4% versus 88.1%, *P*=0.038). Reasons for not achieving device success were different between groups (Figure 2). Patients who were MO had higher rates of severe PPM and mean aortic valve gradients, which remained higher at 1‐year follow‐up. No differences were observed for short‐term mortality (Table 3 and Table S2).

**Table 3:** Clinical End Points and Echocardiographic Data After the Procedure in the Propensity Score–Matched Analysis for Nonobese and Morbidly Obese Cohort

Clinical End Points	BMI 18.5–29.9 (n=770)	BMI >35 (n=770)	*P* Value
In‐hospital mortality	26 (3.4)	28 (3.6)	0.782
In‐hospital or 30‐d mortality	28 (3.6)	35 (4.6)	0.368
Vascular complications			
Major	33 (4.3)	51 (6.6)	0.043
Minor	80 (14.1)	61 (9.3)	0.009
Vascular complications femoral access only			
Major	32 (4.7)	48 (7.2)	0.052
Minor	79 (16.1)	58 (10)	0.003
Major vascular complications femoral access only by closure type			
Percutaneous closure device	22 (4.0)	39 (6.7)	0.046
Surgical cutdown technique	8 (9.3)	9 (15.0)	0.291
Bleeding			
Life‐threatening bleeding	22 (2.9)	19 (2.5)	0.659
Major bleeding	37 (4.8)	44 (5.7)	0.421
Life‐threatening and major	59 (7.7)	63 (8.2)	0.706
Minor bleeding	66 (8.6)	55 (7.3)	0.353
AKI			
Stage I	85 (13.7)	101 (14.8)	0.548
Stage II and III	24 (3.9)	25 (3.7)	0.859
Any stage	109 (17.5)	126 (18.5)	0.646
Coronary occlusion	5 (0.7)	4 (0.5)	0.738*
Periprocedural Stroke	11 (1.4)	12 (1.6)	0.831
Hospital‐acquired pneumonia	11 (1.57)	10 (1.33)	0.690
New permanent pacemaker implantation	91 (13)	108 (15.6)	0.158
Length of hospital stay, d	6 [5–9]	5 [3–8]	<0.001
Post‐TAVR echocardiogram parameters within 30‐d after TAVR			
Moderate‐severe post‐TAVR AR	38 (5.0)	18 (2.5)	0.009
Postprocedural mean aortic valve gradient (mm Hg)	8 [6–11]	10 [7–14]	<0.001
Severe patient‐prosthesis mismatch	7 (1.1)	23 (3.5)	0.004
Device success	678 (88.1)	650 (84.4)	0.038
Echocardiogram parameters at 1‐y after TAVR			
1 y mean aortic valve gradient (mm Hg)	8 [6–11]	10 [7–14]	<0.001

Values are expressed as n (%) or median [IQR]. AKI indicates acute kidney injury; AR, aortic regurgitation; BMI, body mass index; and TAVR, transcatheter aortic valve replacement.

* Fisher’s exact test used.

**Figure 3:**
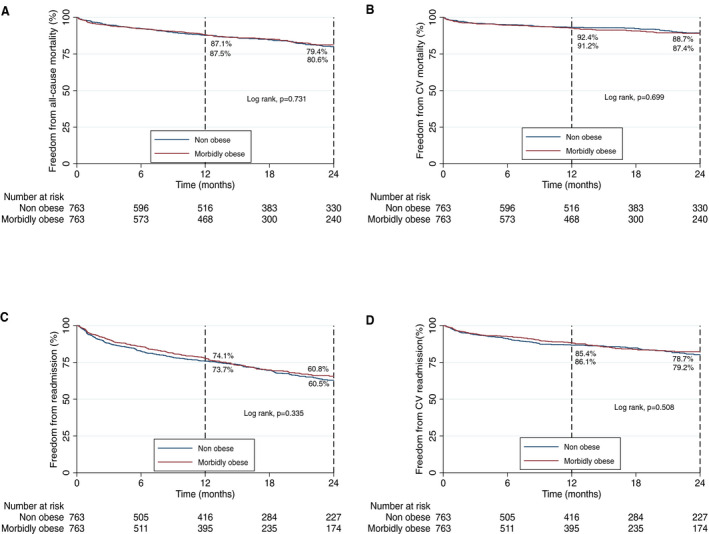
Kaplan‐Meier graph demonstrating 2‐year all‐cause (**A**) and cardiovascular (**B**) mortality and 2‐year all‐cause (**C**) and cardiovascular (**D**) readmission in the propensity score–matched analysis for obese and nonobese groups. CV indicates cardiovascular.

After a median follow‐up of 14.1 months (interquartile range, 6.5–36.0), survival analysis in the matched cohort demonstrated similar rates of freedom from all‐cause and cardiovascular mortality for MO and nonobese groups (79.4 versus 80.6%, *P*=0.731; and 88.7 versus 87.4%, *P*=0.699, respectively) (Figure 3A and 3B). All‐cause and cardiovascular readmission rates at 24 months were high overall, with no difference between groups (Figures 3C and 3D). Figure S3 depicts outcomes in the unmatched cohort.

### Predictors of Outcome in Patients Who Are MO

Table S3 lists predictors of mortality in the whole cohort, while independent predictors of 2‐year mortality in the MO population (n=910) are shown in Table 4. Decreased baseline estimated glomerular filtration rate (hazard ratio [HR], 1.16; 95% CI, 1.07–1.26; *P*<0.001) and nontransfemoral vascular access (HR, 1.82; 95% CI, 1.19–2.79; *P*=0.006) were both independent predictors of all‐cause mortality at 2 years. Neither BMI, body surface area, nor severe PPM predicted 2‐year mortality in the whole or MO cohorts.

**Table 4:** Univariable and Multivariable Analysis of All‐Cause Mortality at 2 Years in Patients With Morbid Obesity (n=910)

	Univariable Analysis HR (95% CI)	*P* Value	Multivariable Analysis HR (95% CI)	*P* Value
COPD	1.44 (1.01–2.07)	0.047		
eGFR per 10 mL/min per 1.73m^2^ decrease	1.15 (1.06–1.25)	0.001	1.16 (1.07–1.26)	<0.001
Baseline hemoglobin*	1.32 (1.06–1.64)	0.013		
Major vascular complication	2.01 (1.51–2.68)	<0.001		
Life‐threatening bleeding or major bleeding	2.66 (1.69–4.18)	<0.001		
Nontransfemoral access	1.70 (1.37–2.10)	<0.001	1.82 (1.19–2.79)	0.006
General anesthesia	1.40 (0.98–1.98)	0.062		
AKI stage II‐III	3.94 (2.16–7.17)	<0.001		
Periprocedure stroke	4.92 (2.30–10.56)	<0.001		

AKI indicates acute kidney injury; COPD, chronic obstructive pulmonary disease; eGFR, estimated glomerular filtration rate; and HR, hazard ratio.

* For every 2‐g decrease.

### Adipose Tissue Distribution Subanalysis

Of 394 CT scans collected that included third lumbar spine images, 275 (69.8%) were suitable for abdominal VAT, IMAT, and SMA analysis. Excessively narrowed field of view (n=85) and asymmetry (n=34) were the main reasons for excluding CT scans. A further 56 patients were excluded from SAT analysis due to an excessively narrowed field of view resulting in 219 SAT analyses being performed. The mean area for each abdominal adipose tissue compartment indexed to body surface area were iVAT 146.0±53 cm^2^/m^2^, indexed SAT 172.4±56.3 cm^2^/m^2^, and indexed IMAT 16.6±8.7 cm^2^/m^2^. The average indexed SMA was 60.2±13.5 cm^2^/m^2^. The proportion of IMAT inside the skeletal muscles was 22±10%. A total of 376 contrast cardiac CT scans were analyzed, and epicardial fat volume quantification was feasible in 266 (70.74%). Reasons to exclude were excessively narrowed field (n=53) or difficulty in identifying the pericardial outline (n=59). The average indexed EAT volume was 47.0±23.1 cm^3^/m^2^. Sarcopenic obesity was found in 7.84% of the population using predefined sex‐specific cutoffs. Absolute and indexed areas and volumes of adipose and muscle tissue analysis are summarized in Table S4.

**Figure 4:**
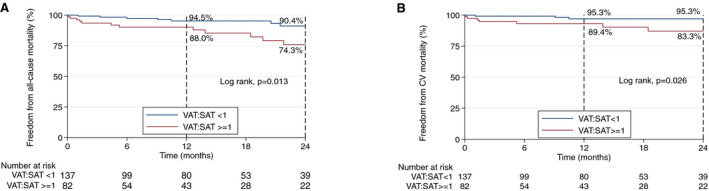
Kaplan‐Meier graphs demonstrating 2‐y all cause (**A**) and cardiovascular (**B**) mortality for patients with visceral to subcutaneous adipose tissue (VAT:SAT) ratio <1 and ≥1. CV indicates cardiovascular; SAT, subcutaneous adipose tissue; and VAT, visceral adipose tissue.

Most periprocedural events were not associated with adipose tissue distribution parameters assessed, except for periprocedural cerebrovascular accident, the risk of which increased with increasing iVAT (odds ratio, 2.09 for each 40 cm^2^/m^2^ incremental increase; 95% CI, 1.05–4.15; *P*=0.036). The optimal cutoff for predicting periprocedural cerebrovascular events was an iVAT of 221.3cm^2^/m^2^, giving an area under the curve of 0.802 (95% CI, 0.4949–1.000) and a sensitivity of 66.67% and specificity of 91.91%. Indexed IMAT and indexed SMA were not associated with periprocedural or midterm outcomes, and no association was found between CT‐defined sarcopenic obesity and 2‐year mortality. An increased risk of all‐cause death at 2 years was found for each 10cm^3^/m^2^ increment in indexed epicardial adipose tissue (HR, 1.16; 95% CI, 1.03–1.30; *P*=0.011) and each 40 cm^2^/m^2^ incremental increase in iVAT (HR, 1.40; 95% CI, 1.05–1.86; *P*=0.021). Furthermore, the proportion of visceral to subcutaneous fat was associated with 2‐year mortality: a ratio of VAT:SAT ≥1 demonstrated the best cutoff point for identifying patients who were MO with an unfavorable obesity phenotype for 2‐year mortality with and an area under the receiver operating characteristic curve of 0.696, giving a sensitivity of 63.7% and specificity of 71.1%. This cut point gave an HR for all‐cause mortality of 3.06 (95% CI, 1.20–7.77; *P*=0.019), cardiovascular mortality of 4.11 (95% CI, 1.06–15.90; *P*=0.041), and readmission of 1.81 (95% CI, 1.07–3.07; *P*=0.027) at 2 years. Figure 1 depicts the adipose tissue distribution analysis of a favorable and unfavorable obesity phenotype. Kaplan‐Meier curves for 2‐year all‐cause and cardiovascular mortality of patients with a VAT:SAT ratio <1 and ≥1 are depicted in Figure 4. After adjustment for other factors associated with mortality, a VAT:SAT ratio ≥1 remained a strong predictor of 2‐year mortality with an HR of 2.78 (95% CI, 1.07–7.19; *P*=0.035). Differences in baseline characteristics in patients with a VAT:SAT ratio <1 and ≥1 are shown in Table S5.

## Discussion

The main findings of the study include the following: (1) TAVR in patients who are MO is a safe procedure with similar periprocedural complications to a cohort of nonobese patients, except for a higher rate of MVCs; (2) device success was lower in the MO cohort, mainly driven by a higher rate of elevated mean aortic gradient and severe PPM, but severe PPM was not a predictor of 2‐year mortality in the MO group; (3) similar 2‐year outcomes were observed in the unmatched and matched MO and nonobese groups; and (4) adipose tissue distribution analysis obtained from the baseline CT scan identified an obesity phenotype (VAT:SAT ratio ≥1) at greater risk of all‐cause and cardiovascular mortality and readmission at 2 years. Our conclusions for this study are supported by a large study population, a robust methodology used to measure adipose tissue distribution, midterm follow‐up, and use of propensity score matching as part of the statistical analysis.

Short‐ and midterm survival was not different between groups in either the unmatched or matched population in our study. These findings suggest that patients who are MO are equally likely to benefit from a TAVR procedure than their nonobese counterparts. However, it does not support the notion of an “obesity paradox” and is more in keeping with prior studies on populations such as ours^7^ using BMI ≥35 kg/m^2^ and with other studies investigating the impact of BMI on outcomes, which showed no difference in mortality rates between obese and normal‐weight patients.^26^, ^27^ Variations in reported results in studies to date examining the impact of BMI on outcomes in TAVR may be related to the use of a single BMI cutoff for obesity (generally >30 kg/m^2^), which fails to take account of varying obesity phenotypes and may lack sensitivity to discern differences in outcomes between obesity subgroups.^15^ Our study concentrated on the MO subgroup, who unlike the moderately obese cohort (BMI, 30–35 kg/m^2^) often have adverse risk factor profiles, and, despite being commonly encountered in real‐world practice, are often underrepresented in clinical trials. However, with comparable outcomes to the nonobese group, our findings suggest that TAVR is a viable option in this population and should be offered to patients who are MO with severe aortic stenosis.

Some differences in valve hemodynamics were noted between groups, which affected device success rate. Increased rate of PPM in both surgical AVR and TAVR has been previously documented in obese patients.^28^, ^29^, ^30^ Likewise, mean aortic gradient and severe PPM was more common in the MO group, despite using larger valve sizes. These findings suggest that careful consideration must be given to choose a prosthesis with a favorable hemodynamic profile to optimize results in the MO population. Surprisingly, we found a decreased rate of moderate to severe aortic regurgitation after TAVR in the MO group (in both the matched and unmatched populations), in accordance with Sharma et al.^7^ Whether this could be related to poorer visualization of regurgitant aortic flow or additional factors that could influence paravalvular leak should be further investigated in future dedicated studies.

Similar to the nonobese group, 20% of the MO cohort died within 2 years, making careful patient selection to avoid futile interventions an important issue for this population. Multivariable analysis highlighted a number of predictive factors among the MO population for 2‐year mortality, which importantly included nontransfemoral access, suggesting transfemoral route should be the preferred approach in this cohort, as in others.^4^ Notwithstanding the preference for the transfemoral route, MVCs were higher in the MO group, including in those who had a transfemoral TAVR performed, a finding also reported by González Ferreiro et al^14^ and previously found by Hibbert et al^31^ in the context of femoral access coronary angiography in patients with extreme obesity. Performing safer percutaneous procedures in patients who are MO requires vigilance when dealing with vascular access. Although more MVCs were seen in the MO group who had a purely percutaneous approach with a closure device, this method had fewer MVCs than those who underwent surgical cutdown for femoral vascular access. A recent study by Kotronias et al^32^ demonstrated a significant reduction in vascular access complications with the use of ultrasound‐guided access. Further studies investigating the importance of preventive strategies such as contralateral vascular protection for percutaneous access in this challenging population are currently awaited (Junquera et al, under review).

### Obesity Phenotype

Adipose tissue distribution analysis can further discern a patient’s obesity phenotype and has the potential to add another dimension to the risk stratification of patients. Visceral adiposity has been found to be associated with development of cardiovascular risk factors and adverse cardiac outcomes, particularly in relation to coronary artery disease,^33^, ^34^ and improves risk prediction for cardiovascular events beyond that of BMI alone.^33^ Its association with outcomes in a MO TAVR population has not been previously described, although some small studies in non‐MO TAVR populations exist.^35^, ^36^ Our study found an increased risk of all‐cause mortality at 2 years with increasing iVAT, which is consistent with the findings of Mancio et al,^35^ who also found high levels of iVAT to be associated with all‐cause mortality in obese (n=44), but not normal weight patients. The large number of patients included in our study with iVAT assessment adds considerable weight to this finding. Importantly, we found that the proportion of visceral to subcutaneous fat had a strong association with readmission and midterm all‐cause and cardiovascular mortality with a VAT:SAT ratio ≥1 resulting in a more than 3‐fold increased risk of all‐cause, and 4‐fold increased risk of cardiovascular death at 2 years. Furthermore, this association with all‐cause mortality was maintained when the VAT:SAT ratio was added to our multivariable analysis. This was despite very similar baseline characteristics in patients with a VAT:SAT ratio >1 and <1 (Table S5), suggesting an added benefit for this ratio in predicting outcomes beyond normal cardiovascular risk factors or indeed BMI. Visceral fat, an ectopic fat deposit, is associated with dysregulated fat metabolism and insulin resistance and is known to be a highly metabolically active tissue,^37^ while abdominal subcutaneous fat has been shown to have an inverse relationship with atherosclerotic disease^38^ and, in the context of TAVR, an inverse relationship with mortality.^36^ Furthermore, increasing VAT:SAT ratio has previously been shown to be an independent predictor of cardiac events in patients referred for CT coronary angiography.^39^ It is unsurprising, therefore, that VAT:SAT ratio in our study demonstrated a strong relationship with midterm outcomes. The similar association with percentage VAT and 2‐year mortality further reinforces the notion that adipose tissue distribution, rather than purely BMI, contributes to the patient’s overall risk. Although VAT:SAT ratio did not correlate with periprocedural events, its predictive power for midterm outcomes serves to provide an opportunity to initiate interventions after TAVR, aimed at reducing overall risk, such as a cardiac rehabilitation program. Whether this could improve mid‐ and longer‐term outcomes should be a new focus for investigation.

Epicardial adipose tissue, another form of ectopic visceral fat deposition, also predicted all‐cause mortality at 2 years. Epicardial fat is thought to have a local, rather than systemic, endocrine effect^25^ and has been associated with coronary calcification and significant coronary artery stenosis, although it has been suggested that this association is stronger in nonobese populations.^40^ Several studies have reported an association with prevalent cardiovascular disease and with major cardiovascular events,^25^, ^41^ consistent with our findings. A high VAT:SAT ratio, high‐percentage VAT, and high epicardial fat represent an adverse metabolic phenotype that has increasingly become of interest.^42^

### Clinical Applicability

From a patient‐centered perspective, the results of our study may contribute to better decision making at the time of planning TAVR. With BMI not predicting midterm outcomes in our cohort, assessment of a patient’s obesity phenotype using the pre‐TAVR CT scan may be a more useful tool to further risk stratify patients who are MO undergoing TAVR. Furthermore, these parameters are easily and quickly derived from the pre‐TAVR CT with many commercially available software packages providing semiautomatic body composition analysis. As a VAT:SAT ratio ≥1 identifies worse patient outcomes resulting from cardiac and noncardiac causes, diligent action in modifying associated comorbidities should be taken. These include strictly controlling cardiovascular risk factors, monitoring patients for endocrine dysfunction, and implementation of dedicated weight‐loss strategies.^43^

### Limitations

Although our study represents a large multicenter analysis of the impact of morbid obesity on outcomes following TAVR, a number of limitations must be acknowledged. First, this is a retrospective analysis of prospectively collected data and, as such, has limitations inherent to this study design. Second, although propensity matching attempts to compensate for the lack of randomization and minimize baseline differences between groups, a number of differences remained, and the possibility of unidentified confounders cannot be excluded. Third, a number of TAVR CT scans were unsuitable for analysis mainly due to narrowed field of view, which may have impacted on our findings relating to the body composition analysis performed. Still, this is the largest CT analysis to date in the TAVR field regarding this subgroup of patients who are MO.

## Conclusions

TAVR in patients who are MO is a safe procedure and demonstrates similar short‐ and midterm outcomes in comparison with nonobese patients, despite slightly higher vascular complications and lower device success rates. Adverse obesity phenotype is an important predictor of midterm outcomes and body composition analysis performed on baseline pre‐TAVR CT can further risk stratify patients beyond baseline BMI.

## Sources of Funding

This study was supported by Fundación Interhospitalaria para la Investigación Cardiovascular (FIC Foundation) via an unrestricted grant from Abbott.

## Disclosures

Dr. Toggweiler is a proctor and consultant for Boston Scientific, Medtronic and Biosensors/New Valve Technology, a proctor for Abbott Vascular, a consultant for Shockwave, Teleflex, Medira, AtHeart and holds equity in Hi‐D Imaging. Dr. Dabrowski is a proctor and consultant for Boston Scientific, has received speaker’s honoraria from Boston Scientific, Edwards Lifesciences Inc. and Medtronic.

Dr Regueiro is a proctor for Abbott. Dr Ribeiro is consultant for Edwards Lifesciences, Medtronic, and Boston Scientific. Dr Garcia del Blanco is proctor for Edwards Lifesciences. Dr Amat‐Santos is a proctor for Boston Scientific. Dr Saia has received speaker’s honoraria from Edwards Lifesciences Inc, Medtronic, Abbott Vascular, and Boston Scientific. Dr Nombela‐Franco has served as a proctor for Abbott and has received speaker’s honoraria from Edwards Lifesciences Inc and Boston Scientific. The remaining authors have no disclosures to report.

## Supporting information

Video S1

Table S1–S5
Figure S1–S3
