# Supplementary material for: Impact of Morbid Obesity and Obesity Phenotype on Outcomes After Transcatheter Aortic Valve Replacement
Source: J Am Heart Assoc. 2021 May 31;10(12):e019051. doi: 10.1161/JAHA.120.019051 (PMC8477858; doi:10.1161/JAHA.120.019051)
Supplement: Supplementary file 2 — Table S1–S5 Figure S1–S3 [file JAH3-10-e019051-s001.pdf]

SUPPLEMENTAL MATERIAL

Table S1. Procedural aspects in unmatched non-obese and morbidly obese cohorts.

	BMI 18.5-29.9 (n= 2,264)	BMI >35 (n= 910)	P
Procedural urgency			
Urgent/Emergent	180 (9.7%)	67 (8.2%)	0.224
Access site			
Transfemoral	1994 (88.1%)	784 (86.2%)	0.139
Non-transfemoral access	270 (11.9%)	126 (13.9%)	0.139
Method of transfemoral access*†			
Percutaneous with closure device	1626 (93.9%)	678 (89.8%)	<0.001
Surgical cut down	105 (6.1%)	77 (10.2%)	<0.001
Prosthesis type			
BEV	899 (39.7%)	432 (47.5%)	<0.001
SEV	1362 (60.2%)	469 (51.5%)	<0.001
Edwards Sapien & XT & S3	899 (39.7%)	432 (47.5%)	<0.001
Medtronic Corevalve, Evolut R, Evolut Pro	1065 (47.0%)	403 (44.3%)	0.159
Other (portico, accurate neo, other)	300 (13.2%)	75 (8.2%)	<0.001
Prosthesis size			
20 – 23 mm	525 (23.5%)	207 (22.9%)	0.782
25 – 27 mm	1005 (45.0%)	394 (44.1)	0.650
29 – 34 mm	702 (31.5%)	293 (32.7%)	0.485
Other procedural aspects			
General anesthesia	958 (42.3%)	350 (38.5%)	0.048
Prior balloon valvuloplasty	1367 (65.9%)	498 (60.1%)	0.003
Balloon post-dilatation	511 (23.6%)	112 (12.5%)	<0.001

*= One center excluded that practices only 'cut down' technique for femoral access
†= femoral access only
BEV: Balloon expandable valve, SEV: Self expanding valve

Table S2. Clinical end points and echocardiographic data post procedure for unmatched non-obese and morbidly obese cohorts.

Clinical endpoints	BMI 18.5-29.9 (n= 2,264)	BMI >35 (n= 910)	p
Mortality			
In-hospital mortality	82 (3.6%)	35 (3.9%)	0.762
In-hospital or 30-day mortality	86 (3.8%)	43 (4.7%)	0.232
Vascular complications			
Major	103 (4.6%)	60 (6.6%)	0.019
Minor	183 (10.3%)	67 (8.7%)	0.196
Vascular complications femoral access only			
Major	96 (4.8%)	55 (7.0%)	0.022
Minor	182 (11.8%)	62 (9.2%)	0.072
Major vascular complications femoral access only by closure method*			
Percutaneous closure device	71 (4.4%)	44 (6.5%)	0.034
Surgical cut-down technique	10 (9.5%)	11 (14.3%)	0.321
Bleeding			
Life-threatening bleeding	69 (3.1%)	23 (2.6%)	0.454
Major bleeding	134 (5.9%)	51 (5.6%)	0.737
Life-threatening and major	203 (9.0%)	74 (8.1%)	0.451
Minor bleeding	217 (9.6%)	61 (6.9%)	0.015
AKI			
Stage I	221 (14.4%)	118 (14.5%)	0.920
Stage II and III	55 (3.6%)	31 (3.8%)	0.769
Any stage	276 (18.0%)	149 (18.4%)	0.814
Coronary occlusion	11 (0.5%)	5 (0.5%)	0.819†
Peri-Procedural Stroke	39 (1.7%)	14 (1.5%)	0.714
Hospital acquired pneumonia	39 (1.9%)	11 (1.2%)	0.236
New permanent pacemaker implantation	228 (11.2%)	121 (14.7%)	0.010
Length of hospital stay, days	6 [5-9]	5 [3-8]	<0.001
Echocardiogram parameters within 30-day post-TAVR			
Moderate-Severe Post TAVR	104 (4.7%)	21 (2.4%)	0.004

AR			
Postprocedural mean aortic valve gradient (mmHg)	8 [6-11]	10 [7-14]	<0.001
Severe patient-prosthesis mismatch	20 (1.1%)	27 (3.5%)	<0.001
Device Success	2031 (89.7%)	759 (83.4%)	<0.001
Echocardiogram parameters at 1-year post TAVR			
Mean aortic valve gradient (mmHg)	8 [5.9-10.8]	10 [7-15]	<0.001

Values are expressed as n (%) or median [IQR]

* One center excluded that practices only ‘cut down’ technique for femoral access

† Fischers exact test used

AR: aortic regurgitation, TAVR: transcatheter aortic valve replacement.

Table S3. Univariable and multivariable analysis of all-cause mortality at 2 years in the whole cohort (non-obese and morbidly obese patients n=3174).

	Univariable analysis HR (95% CI)	p value	Multivariable analysis HR (95% CI)	p value
Morbid obesity	1.01 (0.83-1.25)	0.893		
BMI	0.99 (0.99-1.01)	0.826		
BSA	1.16 (0.81-1.66)	0.430		
Diabetes	1.24 (1.03-1.49)	0.026		
Hypertension	1.32 (1.00-1.74)	0.049		
Smoking	1.25 (1.01-1.55)	0.033		
COPD	1.44 (1.18-1.75)	<0.001	1.38 (1.10-1.74)	0.006
Severe pulmonary hypertension (>55mmHg)	1.49 (1.17-1.90)	0.001		
Peripheral vascular disease	1.59 (1.26-1.99)	<0.001		
Pre-existing atrial fibrillation	1.45 (1.20-1.74)	<0.001		
eGFR <30	2.24 (1.74-2.88)	<0.001		
Baseline Haemoglobin *	1.26 (1.13-1.41)	<0.001		
Moderate-Severe MR	1.29 (1.03-1.61)	0.024	1.35 (1.05-1.75)	0.022
Urgent/Emergent procedure	1.73 (1.29-2.33)	<0.001		
Non-transfemoral access	1.66 (1.32-2.09)	<0.001	1.51 (1.16-1.97)	0.002
Conversion to surgery	6.06 (2.87-12.79)	<0.001		
Hospital acquired pneumonia	3.85 (2.56-5.82)	<0.001		
Major vascular complications	1.97 (1.43-2.72)	<0.001		
Life threatening or major bleeding	2.31 (1.80-2.96)	<0.001	1.86 (1.39-2.48)	<0.001
Blood transfusion	1.69 (1.39-2.07)	<0.001		
Periprocedural CVA	3.49 (2.27-5.35)	<0.001		
New onset atrial fibrillation	1.33 (1.00-1.766)	0.051		
Post procedure moderate-severe AR	1.79 (1.13-2.84)	0.013		
In-hospital days	1.01 (1.01-1.02)	<0.001		
AKI stage II-III	4.65 (3.31-6.54)	<0.001	3.88 (2.72-5.53)	<0.001

AKI: acute kidney injury, AR: aortic regurgitation, BMI: body mass index, BSA: body surface area, COPD: Chronic obstructive pulmonary disease, CVA: cerebrovascular accident, eGFR: estimated glomerular filtration rate, MR: mitral regurgitation.

*For every 2gram decrease

Table S4. Summary of body composition analysis.

Body composition component	Mean (SD)
SAT area cm^2	357.9 (118.3)
iSAT area cm^2/m^2	172.4 (56.3)
VAT area cm^2	311.3 (128.6)
iVAT area cm^2/m^2	146.0 (53.0)
VAT:SAT	1.03 (0.77)
Percentage Visceral adipose tissue	46 (13)
IMAT cm^2	35.2 (19.0)
iIMAT (indexed intramuscular adipose tissue) cm^2/m^2	16.6 (8.7)
Percentage fatty muscle (%)	22.0 (10.0)
IMAT:SMA	0.30 (0.19)
SMA cm^2	127.3 (33.7)
iSMA area cm^2/m^2	60.2 (13.5)
Sarcopenic obesity*	7.84%
EAT volume cm^3	98.3 (50.4)
iEAT cm^3/m^2	47.0 (23.1)

Values are presented as mean and standard deviation (SD). Indexed values are indexed to body surface area (BSA)

*Sarcopenic obesity defined as height indexed skeletal muscle area (hiSMA) ≤ 38.5cm2/m2 for females and ≤ 52.5cm2/m2 for males

EAT: epicardial adipose tissue, iEAT: indexed epicardial adipose tissue, IAT: Intramuscular adipose tissue, iIMAT: indexed intramuscular adipose tissue, IMAT:SMA ratio of intramuscular fat area to skeletal muscle area, SAT: subcutaneous adipose tissue, iSAT: indexed subcutaneous fat, SMA: skeletal muscle area, iSMA: indexed skeletal muscle area, VAT: Visceral adipose tissue, iVAT: indexed visceral adipose tissue, VAT:SAT: ratio of visceral adipose tissue area to subcutaneous adipose tissue area.

Table S5. Baseline characteristics in morbidly obese cohort according to VAT:SAT ratio.

	**VAT:SAT ratio <1 (n= 137)**	**VAT:SAT ratio ≥1 (n= 82)**	**p**
Age, years	77.4 (7.34)	77.3 (6.7)	0.892
Female sex	118 (86.1%)	18 (23.2%)	<0.001
Body mass index, kg/m^2	39.0 (3.9)	38.5 (3.03)	0.323
Diabetes mellitus	75 (54.74%)	53 (64.63%)	0.151
-Insulin use	27 (37.0%)	29 (56.9%)	0.029
Hypertension	123 (89.8%)	78 (95.1%)	0.164
Hyperlipidaemia	102 (74.5%)	58 (73.4%)	0.867
Baseline creatinine (mg/dL)	1.09 (0.46)	1.40 (0.80)	<0.001
eGFR <30 $ml/min/1.73m^2$	8 (5.8%)	9 (11.0%)	0.196*
Coronary artery disease	58 (42.3%)	46 (56.1%)	0.048
Pre valve surgery	9 (6.6%)	4 (4.9%)	0.608
Valve in Valve TAVR	6 (5.2%)	2 (2.7%)	0.468*
Atrial fibrillation	45 (32.9%)	26 (31.7%)	0.862
Previous pacemaker	13 (9.5%)	10 (12.2%)	0.649
COPD	35 (25.6%)	28 (34.1%)	0.174
Previous cerebrovascular accident	11 (8.0%)	8 (9.8%)	0.660
Peripheral vascular disease	16 (11.7%)	15 (18.3%)	0.174
Baseline hemoglobin (g/dL)	11.9 (1.6)	12.2 (1.8)	0.297
NT-Pro BNP (median, IQR)	877.45 [340 -1897]	887.5 [273-1677.5]	0.981
Logistic EuroSCORE [median IQR]	11.63 [7.05-17.02]	11.2 [8.12-17.0]	0.741
EuroSCORE II [median IQR]	2.97 [1.94-5.52]	4.56 [2.42-6.96]	0.026

STS [median IQR]	4.05 [2.9-6.71]	4.1 [3.01-6.40]	0.968
Moderate or severe PHT	57 (47.9%)	36 (54.6%)	0.386

Values are expressed as n (%) or median [IQR]

*Fischer exact test used

eGFR: estimated glomerular filtration rate, PHT: pulmonary hypertension, TAVR: transcatheter aortic valve replacement

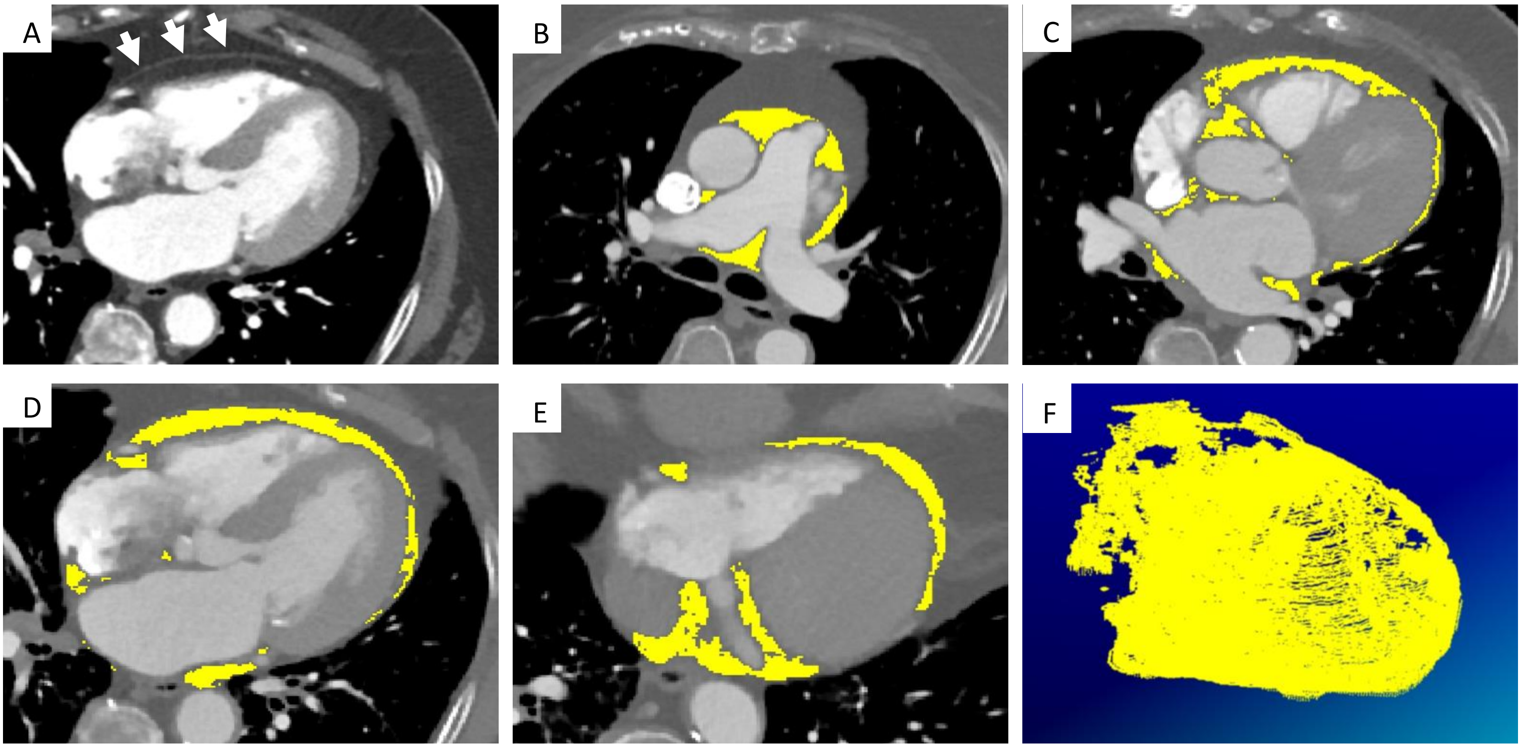

Figure S1. Typical cardiac CT, with contrast, demonstrating the fibrous pericardium (A, white arrows) and subsequent segmentation of epicardial adipose tissue (EAT) within the fibrous pericardium. Using a software package, EAT was segmented based on a Hounsfields unit thresholds of -190 to -30 from the bifurcation of the pulmonary artery (B) continuing to the diaphragm. Images C-E represent examples of slices taken from the mid atrial level, mid-ventricular 4-chamber and lower ventricular 4 chamber view respectively. Epicardial adipose tissue was manually redefined every 3 slices in order to correct contours and avoid inclusion of paracardial adipose tissue (outside the pericardial sac). The software calculated the EAT volume (cm^3) by summing the EAT area in each slice and taking into account slice thickness and intersection gap**.** Image F represents a 3D reconstruction of the EAT in this patient (a female with BMI 35.4kg/m^2 and EAT volume of 108.6 cm^3 and iEAT of 56.77 cm^3/m^2).

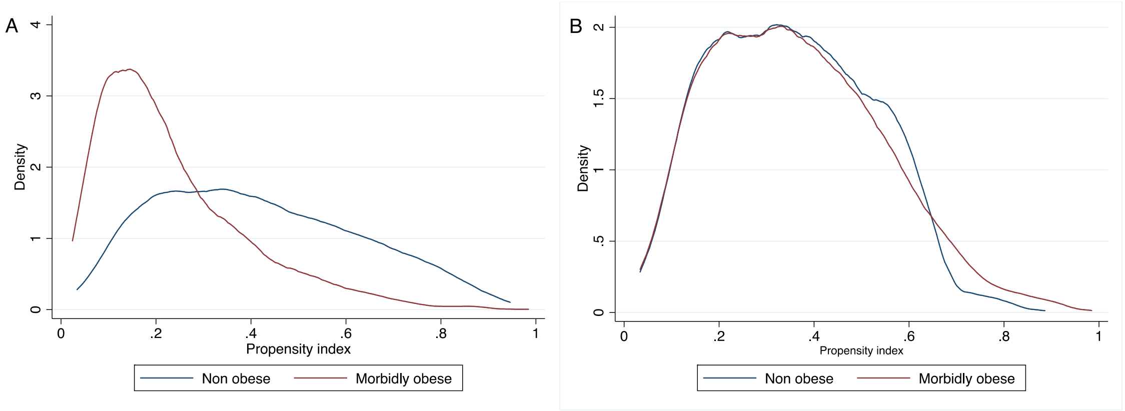

Figure S2. Density plots showing the density index pre- (A) and post- (B) propensity-score matching.

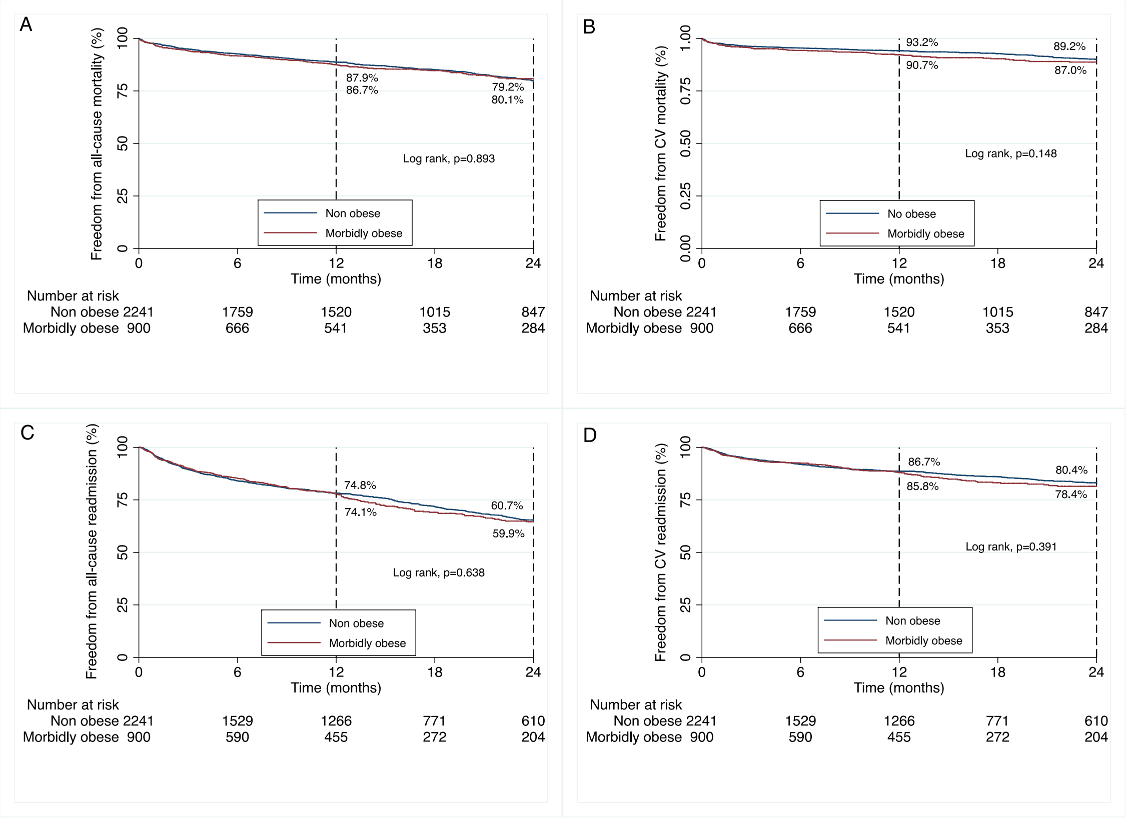

Figure S3. Kaplan Meier graph demonstrating 2-year all-cause (A) and cardiovascular (B) mortality and 2-year all-cause (C) and cardiovascular (D) readmissions for non-obese and morbidly obese groups in the unmatched cohort.

Supplemental Video Legend:

Video S1. 3D reconstruction of epicardial fat segmentation. The video demonstrates assessment of epicardial fat from the bifurcation of the pulmonary artery to the diaphragm. Best viewed with Windows Media Player.
